# Elevated Serum SIRT 2 May Differentiate Parkinson’s Disease From Atypical Parkinsonian Syndromes

**DOI:** 10.3389/fnmol.2019.00129

**Published:** 2019-06-12

**Authors:** Amrendra Pratap Singh, G. Ramana, Teena Bajaj, Vishwajeet Singh, Sadanand Dwivedi, Madhuri Behari, A. B. Dey, Sharmistha Dey

**Affiliations:** ^1^Department of Biophysics, All India Institute of Medical Sciences, New Delhi, India; ^2^Department of Medicine, All India Institute of Medical Sciences, New Delhi, India; ^3^Department of Biostatistics, All India Institute of Medical Sciences, New Delhi, India; ^4^Department of Neurology, All India Institute of Medical Sciences, New Delhi, India; ^5^Department of Geriatric Medicine, All India Institute of Medical Sciences, New Delhi, India

**Keywords:** Parkinson’s disease, atypical parkinsonian syndromes, protein marker, α-synuclein, sirtuin, SPR

## Abstract

Atypical Parkinson syndromes (APSs) often have symptoms that overlap with those of Parkinson’s disease (PD), especially early in the disease, making these disorders difficult to diagnose. Previous studies have demonstrated an association of oligomeric α-synuclein (α-Syn), a key element in the pathogenesis of PD, with Sirtuin (SIRT)2 proteins for modulating PD. We aimed to evaluate SIRT protein expression in serum of PD patients and compare it with APSs and normal elderly control (GC) and to correlate this with α-Syn. SIRT protein expression was evaluated in sera of 68 PD; 34 APS and 68 GC without any neuro-psychiatric illness as controls by surface plasmon resonance (SPR). SIRT2 expression was correlated with α-Syn in PD and GC. Significant (*p* < 0.0001) differences were observed between serum SIRT2 concentration in PD and APS and GC as well as between APS and GC. Receiver operating characteristic (ROC) analysis revealed the strong cut-off value to differentiate PD from APS and GC and also APS from GC. Significant correlation was observed among SIRT2 levels in early PD patients with Unified Parkinson’s Disease Rating Scale (UPDRS), Hoehn & Yahr (H & Y) and increased duration of disease. In addition, a strong positive correlation of SIRT2 with α-Syn (*p* < 0.0001) was observed. However, no such difference was detected for serum SIRT1 in cases of PD and APS or for GC. The present study is the first to report elevated serum SIRT2 in PD. The study also provided a simple test to distinguish PD from APS and may have translational utility for diagnosis.

## Introduction

Parkinson’s disease (PD) is a common movement disorder of the aging population. It is the second most common neurodegenerative disorder after Alzheimer’s disease. It affects 1–2 per 1,000 of the population and its prevalence increases with age to affect 1% of the population above 60 years of age (Tysnes and Storstein, 2017). The pathological changes in PD are well established as being the loss of dopaminergic neurons in the substantia nigra and the presence of cytoplasmic inclusions of Lewy bodies (LBs). LBs primarily consist of α-synuclein protein (α-Syn). α-Syn is primarily expressed by neurons and constitutively released into the extracellular space (Stefanis, 2012). The N-terminal acetylation of α-Syn significantly reduces oligomerization of the protein and thus disrupts intermolecular H-bonds, thereby slowing the initiation of the process (Bu et al., 2017). Recently, it was found that Sirtuin-2 (SIRT2) deacetylates α-Syn (de Oliveira et al., 2017). Sirtuins are NAD^+^-dependent protein deacetylases that mediate calorie restriction and promote longevity. The overexpression of SIRT1 reduces the aggregation of α-Syn in SHSY-5Y cells (Singh et al., 2017). SIRT2 deacetylates α-tubulin, an important pathway involved in the neurodegeneration of PD, promoting the buildup of its toxic complex with α-Syn oligomers (Kazantsev and Kolchinsky, 2008). Several reports have supported this observation by the inhibition of SIRT2 and reduction in the formation of the toxic complex and neurotoxicity (Outeiro et al., 2007; Chen et al., 2015). The inactivation of SIRT2 results in a rescue effect similar to that observed in the activation of SIRT1. Hence, it would be useful to examine the expression of SIRT1 and SIRT2 in PD in view of their opposite roles in the same cycle of α-Syn.

The main symptoms of PD are tremor, gait disturbance and movement. The diagnosis of PD is often difficult, especially at early stage due to overlapping symptoms. Phenotypically similar Atypical Parkinsonian Syndromes (APSs)—namely multiple system atrophy (MSA) and progressive supranuclear palsy (PSP)—can pose diagnostic challenges, even though the pathogenesis and treatment response are dissimilar. No gold standard diagnostic tests for PD are related to the underlying biological process, which necessitates research on biomarker development.

In view of the need for diagnostic tests with biological relevance in pathogenesis that can be performed easily, blood-based biomarkers have caught the imagination of researchers (Baird et al., 2015).

This study quantified SIRT protein levels using the real time label-free surface plasmon resonance (SPR) technique (Kim et al., 2006), which is well established for biomarker discovery in the picomolar range. This study evaluates the level of SIRT1 and SIRT2 protein expression in the serum of PD patients and compares it with APSs and normal elderly controls (GC) in addition to showing the correlation with serum α-Syn levels.

## Materials and Methods

### Study Participants

One-hundred and two patients with PD and similar movement disorders and various stages of disease progression were recruited from the Department of Neurology and Geriatric Medicine of the All India Institute of Medical Sciences, New Delhi, India. Diagnosis of PD, PSP and MSA was carried out as per established clinical criteria by way of the UK Parkinson’s Disease Society Brain Bank Criteria for PD (Hughes et al., 1992), NINDS criteria for PSP (Litvan et al., 1996) and consensus criterion for MSA (Osaki et al., 2009). There were 68 cases of PD and 34 cases of APS (20 MSA and 12 PSP) in the sample. Among these PD patients, 22 with PD whose disease duration was ≤3 years were considered as the early stage cohort as was reported earlier by Hansson et al. (2017). Sixty-eight individuals over 60 years of age with good health (no chronic disease like hypertension, diabetes and no history of any disorder of the central nervous system) were invited for participation in the study through advertisements or general announcements at the hospital.

Demographic details, duration of symptoms up to recruitment to the study, age at onset of illness, family history and co-morbidities were recorded. Detailed clinical assessment for Parkinson’s Disease using the Unified PD Rating Scale (UPDRS, part III motor score) and Hoehn & Yahr staging systems was carried out. Venous blood (2 mL) was collected from the participants in yellow vacutainer (Vacutainer^®^ SST^TM^ Tubes) and was allowed to settle for 1 h at room temperature. The buffy coat was removed from the blood and centrifuged at 3,000 rpm for 20 min. Serum was collected and stored at 70°C in multiple aliquots.

Ethical Statement: The study was approved by the Ethics Committee, All India Institute of Medical Sciences, New Delhi, India (IECNP-221/05.06.2015, RP-01/2015) and written informed consent was obtained from each participant.

### Estimation of Serum Sirtuins and α-Syn

All SPR measurements were performed at 25°C using the BIAcore-3000 (Wipro GE Healthcare, Upasala, Sweden), which is a biosensor-based system for real-time label-free specific interaction analysis. SPR is one of the most powerful technologies to determine specificity, affinity and kinetic parameters during the binding of macromolecules, including protein-protein, protein-DNA, enzyme-substrate or inhibitor, receptor-drug, lipid membrane-protein and protein-polysaccharide interactions. This technique measures the refractive index changes in the vicinity of thin metal layers in response to bimolecular interactions and the real-time response of the experiment is usually presented in the form of a sensorgram.

Mouse anti-SIRT1 IgG (Santa Cruz Bio-technology, Santa Cruz, CA, USA), goat anti-SIRT2 IgG (Santa Cruz Bio-technology, Santa Cruz, CA, USA) and mouse anti-α-syn IgG (Santa Cruz Bio-technology, CA, USA) were used for immobilization on different flow cells of a CM5 sensor chip using the amine coupling kit (Wipro GE Healthcare, Upasala, Sweden). The system was equilibrated with running buffer, i.e., HBS-EP buffer (Wipro GE Healthcare, Upasala, Sweden), and maintained at a flow rate of 5 μL/min. The experimental flow cell dextran matrix was activated using a 1:1 volume mixture of N-ethyl-N′-3-diethylaminopropylcarbodiimide (EDC; 75 μg/μl) and N-hydroxysuccinimide (NHS; 11.5 μg/μl). All three antibodies (100 μg/mL) were diluted with 10 mM sodium acetate (pH 5) and injected over the activated chip surface on three different flow cells, respectively. Unreacted groups were blocked by ethanolamine (pH 8.5).

Standard graphs were prepared by passing different known concentrations of pure recombinant SIRT1 proteins (0.7, 3.5, 7, 10.5, 14, 17.5, 21, 28 ng/μL), SIRT2 proteins (1.7, 8.5, 17, 25.5, 34, 42.5, 51, 68 ng/μL) and α-syn proteins (5, 10, 20, 40, 80, 160, 320 ng/μL) over corresponding antibody on the respective flow cells of sensor chips, and response unit (RU) values were recorded. Recombinant SIRT1, SIRT2 and α-Syn proteins were cloned, expressed and purified in bacterial systems. Serum samples were diluted (1:70) with HBS-EP buffer, passed over immobilized antibodies and RU was obtained for each sample. Each sample was analyzed in triplicate. The concentrations of SIRT1, SIRT2 and the α-Syn of study groups were determined from respective standard curves by extra polating different RU values obtained for each sample.

### By Western Blot

Serum samples collected from the PD, APS and GC groups were subjected to removal of major interfering proteins using an Albumin OUT kit according to the manufacturer’s protocol (G-Biosciences, St. Louis, MO, USA). Total protein concentration was determined using a bicinchoninic acid assay (BCA) with bovine serum albumin as the standard. Total protein (40 μg) was separated by sodium dodecyl sulfate polyacrylamide gel electrophoresis (SDS-PAGE). After electrophoresis, proteins were transferred to polyvinylidenedifluoride (PVDF) membranes (MDI Membrane Technologies, India). The membranes were then blocked over BSA 5% w/v with 0.05% Tween-20 prepared in TBS (10 mm Tris pH 7.5, 150 mm NaCl) for 2 h and subsequently incubated with primary antibodies diluted with TBS at 4°C overnight. The following antibodies and titers were used: mouse anti-human SIRT1 IgG (1:300) and goat anti-human SIRT2 IgG (1:300). After washing with TBS-T (20 mm Tris pH 7.5, 500 mm NaCl, 0.1% Tween-20), membranes were incubated with HRP-conjugated secondary antibodies—goat anti-mouse IgG (1:4,000; Santa Cruz Biotechnology, Santa Cruz, CA, USA) and Donkey Anti-Goat IgG (1:1,000 dilution; Santa Cruz Biotechnology, Santa Cruz, CA, USA)—at room temperature for 1 h. After washing with TBS-T, bands were visualized using SuperSignal West Pico Chemiluminescent substrate (Pierce Biotechnology; Thermo Scientific, Rockford, IL, USA). This experiment was also performed with recombinant α-syn protein to check the specificity of sirtuin (anti-SIRT1 and anti-SIRT2) antibody. Quantification of band intensity was performed using myImageAnalysis software (Thermo Scientific, Rockford, IL, USA).

### Statistical Analysis

Statistical analysis was done using STATA version 14.2 (Stata. Corp. LP, College Station, TX, USA) and GraphPad Prism 5 (La Jolla, CA, USA). All the experiments were done in triplicate and mean ± SD was determined by GraphPad Prism 5. Apart from descriptive statistics, to find the association between qualitative variables, chi square test or Fisher exact test was used. To measure the correlation, Pearson correlation or Spearman rank correlation was used as required. Baseline comparison of quantitative measures between two groups was made using independent *t*-test or the Wilcoxon rank sum test and for more than two groups one-way ANOVA with Bonferroni correction was used. Receiver operating characteristic (ROC) curves were constructed to determine best cut-off, and area under the curve (AUC) was used to compare predictive ability of proteins. The best cut-off point was the point where the sum of specificity and sensitivity were maximized, when equal weight was given to both. A *p*-value less than 0.05 was considered as statistically significant.

## Results

### Demographic Data of the Study Groups

Descriptive statistics of cases and controls are presented in Table 1. Mean age and body mass index (BMI) were similar among cases and controls. Eleven (16.17%) PD cases had positive family history and the rest were sporadic PD. In PD, the mean Hoehn & Yahr stage was 2.15 ± 0.67 with a UPDRS part III motor score of 23.29 ± 10.79.

**Table 1:** Descriptive statistics of the study population.

Variables (N)	GC (68)	PD (68)	APS (34)	*p*-value
Male (%)	51.47	86.76	61.76	0.000^a^
Family History (%)	Nil	16.17	Nil	
Age (years), Mean ± SD	64.76 ± 5.99	65.51 ± 4.71	67.18 ± 6.15	0.2159^b^
Disease duration (years) Mean ± SD	Nil	6.11 ± 4.66	3.92 ± 4.13	0.0058^c^
BMI kg/m^2^, Mean ± SD	23.14 ± 4.30	23.67 ± 3.18	24.69 ± 3.17	0.0919^b^
SIRT1 Mean ± SD (95% CI) ng/μL	6.16 ± 1.13 (5.90–6.44)	5.89 ± 0.70 (5.73–6.07)	5.84 ± 0.90 (5.30–6.40)	OA = 0.3319^b^ GC*PD = 0.099 GC*APS = 0.239 PD*APS = 0.961
SIRT2 Mean ± SD (95% CI) ng/μL	11.18 ± 2.35 (10.62–11.76)	16.19 ± 2.78 (15.52–6.86)	13.86 ± 2.56 (12.97–14.76)	OA = < 0.0001^b^ GC*PD = *p* < 0.0001 GC*APS = *p* < 0.0001 PD*APS = *p* = 0.0001

BMI, body mass index; OA, Overall p value, GC*PD; GC vs. PD, GC*APS; GC vs. APS, PD*APS; PD vs. APS. a, chi square test; b, one-way ANOVA with Bonferroni correction; c, Wilcoxon rank sum test.

### Quantitative Analysis of Serum Sirtuins and α-Syn

#### Estimation by SPR

The SPR signals (RU value) for immobilization of human SIRT1, SIRT2 and α-syn antibodies over sensor chip CM5 were 6,164.6, 6,075.8 and 6,046.4, respectively (Supplementary Figures S1A–C). The binding pattern of the ligands, i.e., pure recombinant SIRT1, SIRT2 and α-Syn proteins, plotted in a standard curve were in the linear range (Supplementary Figures S1D–F).

**Figure 1:**
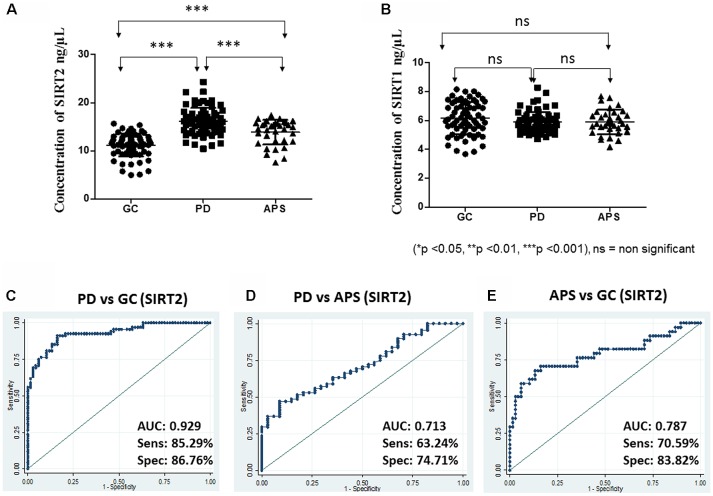
Scatter plot of serum **(A)** Sirtuin 2 (SIRT2) and **(B)** SIRT1 concentration in elderly control (GC), Parkinson’s disease (PD) and atypical parkinson syndrome (APS) by one-way ANOVA with Bonferroni correction. Receiver operating characteristic (ROC) curves of serum SIRT2 between different groups: **(C)** PD vs. GC, **(D)** PD vs. APS and **(E)** APS vs. GC. ***p < 0.001; ns, non-significant.

The concentration of the sirtuins in serum samples were evaluated from the standard curve and observed mean concentration of serum SIRT2 was significantly (*p* < 0.0001) higher in case of PD compared to APS and GC (Table 1, Figure 1A). However, no significant difference existed in the concentration of serum SIRT1 between cases of PD and APS and GC (Figure 1B). Further, no significant (*p* = 0.2389) difference was observed in SIRT1 level between APS and GC (Table 1).

Based on the SPR data, the area under ROC was computed to measure the utility of both SIRT1 and SIRT2 as potential markers for PD between two diagnostic groups to evaluate how well a parameter can distinguish between them. In this study, higher levels were associated with disease condition; hence, the ROC curves were constructed to detect PD. ROC analysis showed that SIRT2 could differentiate PD from GC (AUC = 0.929; 95% CI: 0.89–0.97; cutoff: 13.50 ng/μL) with high sensitivity and specificity (Figure 1C). SIRT2 could differentiate PD from APS with good sensitivity and specificity (AUC = 0.713; 95% CI: 0.61–0.81; cut-off: 15.35 ng/μL; Figure 1D) as well as APS from GC (AUC = 0.787; 95% CI: 0.68–0.89; cut-off: 13.07 ng/μL; Figure 1E). However, SIRT1 did not have the strength to discriminate the disease from GC (AUC = 0.413; 95% CI: 0.31–0.51) and APS (AUC = 0.490; 95% CI: 0.36–0.62). No difference was noted in relation to family history of disease (*p* = 0.4609).

**Figure 2:**
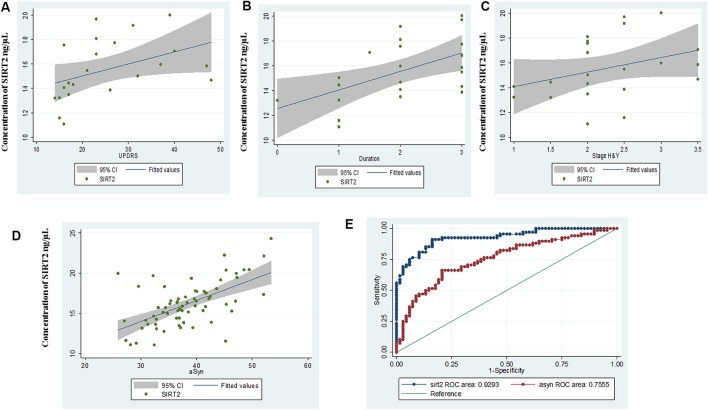
Scatter plot for correlation of serum SIRT2 with **(A)** Unified Parkinson’s Disease Rating Scale III (UPDRS III), **(B)** duration, **(C)** Hoehn & Yahr (H&Y) in early disease group, **(D)** α-synuclein (α-syn) and **(E)** comparative ROC of SIRT2 and α-syn.

In PD patients with early-stage disease duration (≤3 years), a correlation of serum SIRT2 concentration was noted with UPDRS part III motor score (*r* = 0.605, *p* = 0.0028), duration (*r* = 0.54, *p* = 0.0101) and H&Y stage (*r* = 0.41, *p* = 0.05; Figures 2A–C). However, no significant correlation of SIRT2 with UPDRS or H&Y was observed with total PD in the study cohort.

α-Syn serum concentrations showed the same pattern as SIRT2 and were significantly (*p* < 0.0001) elevated in PD (38.34 ± 6.32 ng/μL, 95% CI: 36.81–39.87) as compared to GC (32.92 ± 4.81 ng/μL, 95% CI: 31.75–34.08; Supplementary Figure S2A). AUC of α-Syn from ROC curve was found to be 0.755 (95% CI: 0.67–0.84, cut-off: 35.05 ng/μL; Supplementary Figure S2B). Correlation coefficients indicated a highly positive correlation between α-Syn levels and serum SIRT2 (*r* = 0.723, *p* < 0.0001) within PD patients (Figure 2D). The comparative ROC of SIRT2 and α-Syn is illustrated in Figure 2E, which shows that predictive performance of SIRT2 was significantly better (*p* < 0.0001). Further, we did not observe significant correlation of α-Syn levels with age (*r* = 0.083, *p* = 0.5028), H&Y stage (*r* = 0.109, *p* = 0.376), UPDRSIII score (*r* = 0.2, *p* = 0.101) or disease duration (*r* = 0.1, *p* = 0.405). Also, the levels of serum SIRT1 did not correlate with serum α-Syn levels (*r* = 0.142, *p* = 0.2459) or SIRT2 (*r* = 0.110, *p* = 0.3718).

#### Western Blot

**Figure 3:**
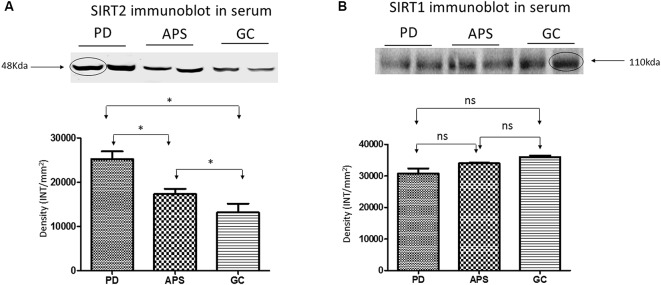
Western blot and density analysis by one-way ANOVA with Bonferroni correction of **(A)** SIRT2 and **(B)** SIRT1 in the serum of PD, APS and GC. Lane 1–2 (PD), Lane 3–4 (APS), Lane 5–6 (GC). Histograms representing normalized mean integrated density ± standard deviation values. *p < 0.05; ns, non-significant.

Western blot analysis of the serum samples was used to validate the differential expression of SIRT1 and SIRT2 in PD, APS and GC. Results were consistent with the band density of SIRT1 and SIRT2 in PD when compared to APS and GC (Figures 3A,B) as demonstrated by SPR data.

## Discussion

In this study, SIRT1 and SIRT2 were estimated for the first time in the serum of PD patients and compared with APSs and GCs. Sporadic PD cases are 85%–90% natural and the rest are familial in origin. In the present study only 16% cases were of familial origin, in line with previous reports (Singleton et al., 2013). Although the present study is not an epidemiological study, the lower number of APSs does reflect their relative rarity compared with PD, as reported earlier (Osaki et al., 2011).

SIRT2 concentration was high in cases of PD and differentiated from APSs and GCs. The level of SIRT2 in PD patients was found to be increased irrespective of the other covariates. This study found a significant correlation of SIRT2 levels in early PD patients (disease duration ≤3 years) with different parameters, such as UPDRS, H & Y and an increased disease duration. Serum SIRT2 level can also differentiate PD from APS patients according to an ROC analysis with efficient cut-off values. However, no significant difference in SIRT1 was observed among the study groups.

Knowledge about the role of sirtuins in complicated biological systems that influence many regulator molecules and pathways in health and disease is still incomplete. Few studies of SIRT proteins in humans are available for neurodegeneration such as PD. In our earlier study with sirtuin in Alzheimer’s disease, which is another of the most common neurodegenerative diseases, we found the downregulation of SIRT1 in serum compared with mild cognitive impairment as well as the control group (Kumar et al., 2013). The present study with PD surprisingly showed no effect on serum SIRT1 and overexpressed SIRT2 compared with the control. Hence, two isoforms of the same protein family are altered in a different manner in two of the most prominent neurodegenerative diseases with very high sensitivity and specific statistical parameters.

The periodic updating of diagnostic criteria reflects the continuity of efforts to understand the disease. It also increases knowledge on the critical pathophysiology of PD to establish a gold standard for diagnosis criteria based on the pathology of the disease state. Ideally, the body fluid closest to the organ involved provides the best indication of the pathological process. However, it may not always be easy to access such body fluid repeatedly, namely CSF, without the risk of harm, especially in older subjects. Thus, the analysis of other body fluid proteomes also appears to be a promising approach to identify the protein biomarkers of diseases. The human protein circulates in the serum due to secretion and leakage from a variety of tissues (Taylor, 1969). It was reported earlier that these circulated proteins reflect human physiological or pathological conditions (Anderson and Anderson, 2002). Blood-based markers offer many advantages due to their easy accessibility compared with CSF. In this study, SPR technology is used for the assay. SPR is an optical phenomenon that is sensitive to changes in the optical parameter of the medium close to a metal surface *via* a quantum mechanical aspect to determine specificity, affinity and kinetic parameters during the study of biomolecular interactions. SPR has proven to be useful in the discovery of biomarkers due to being label-free, its real-time nature, low amount requirement, reproducibility, robustness and high sensitivity. It has been successfully applied in detection and validation of biomarkers for numerous diseases (Cooper, 2003; Homola, 2003; Nguyen et al., 2015). The SPR technique offers several advantages over other immunological experiments such as ELISA for the reusability of the antibody over a large number of samples.

In the present study, circulating α-Syn was found to be overexpressed in PD compared with controls, in line with earlier reports. In recent years, substantial progress has been made in identifying the events, pathways and proteins involved in the pathogenesis of PD. The current research interest focuses on α-Syn, which has been implicated in the development of familial as well as sporadic forms of PD. Both structurally normal as well as abnormal α-Syn have been considered to be involved in the genesis of PD (Stefanis, 2012). The abnormal α-Syn increased the toxicity of brain cells, which can trigger neurodegeneration in critical areas of the brain, leading to the development of the disease, suggesting the possibility of it being a prion protein and PD a prion disease. The variable a-Syn was reported in CSF and blood levels of PD patients by different groups in the literature. Supporting this hypothesis, increased (Aasly et al., 2014) and decreased (Mollenhauer et al., 2011) α-Syn concentrations in CSF have been observed in PD, while normal (Gupta et al., 2015) and upregulated (Duran et al., 2010) α-Syn concentrations were reported in plasma of PD patients.

The linkage between α-Syn and sirtuins in PD is interesting. SIRT1 localized mainly in the nucleus (Tanno et al., 2007) and SIRT2 is localized in the cytoplasm (North et al., 2003). SIRT2 deacetylates α-tubulin, which stabilizes microtubules, and microtubules are found throughout the cytoplasm. The possible hypothesis involved in the pathophysiology of PD may be either the interaction of the deacetylated α-tubulin oligomer by SIRT2 with an α-Syn oligomer or the promoting initiation of α-Syn oligomerization by deacetylating the N-terminal of α-Syn by SIRT2. Hence, SIRT2 might be associated with early events of pathogenesis. SIRT2 is indirectly associated with the cellular processes involved in the pathophysiology of neurodegenerative disorders such as autophagy, oxidative stress and inflammation by deacetylating FOXO1 to mediate autophagy in the context of neurodegeneration (Salih and Brunet, 2008). Previous studies have shown the rescue of neuronal death by the inhibition of SIRT2 in PD models. This may be due to the formation of a larger LB-like inclusion by reducing the deacetylation of α-tubulin (Outeiro et al., 2007). The exact role of SIRT1 and SIRT2 in PD is not yet clear. This result also correlates with the overexpressed α-Syn protein in the serum of PD patients. The elevated level of α-Syn in PD supports previous findings reported in the literature.

PD remains a clinical diagnosis even 200 years after its first description, as no gold standard biomarker has evolved for pre-mortem diagnosis. Thus, with the best of clinical abilities and use of guidelines, deficiencies in diagnosis remain (Rizzo et al., 2016). Being a chronic disease of subtle symptoms and signs, diagnosis is often delayed, and the course of the disease is prolonged with the progression of disease pathology. A small subset of patients with movement disorder disease has extensive involvement of the brain with atypical manifestations and poorer response to treatment. Most PD patients are diagnosed at a late stage when vulnerable dopaminergic neurons in the substantia nigra have already been lost, and it is nearly impossible to detect PD by any screening test before the appearance of motor symptoms. To date, lower uric acid, lower plasma ApoA1 and lower EGF levels have been identified as blood-based biomarkers in independent cohorts of PD patients (Chahine et al., 2014). However, none of these is a potential marker for the early detection of the disease. The proposed protein marker (SIRT2) can be the starting point of establishing diagnostic tests for PD, alone or in combination with other biomarkers.

The strength of the study is the label-free highly sensitive SPR technique used for the quantification of sirtuin proteins. This can detect the binding target molecule in the picomolar range. The technique is very sensitive as the minimum detection limit is 1 pg/mL. This study for the first time reported the serum sirtuin level in PD, APS and GC, which can be used in a panel of biomarkers for the early detection of diseases as well as to differentiate PD from APSs.

The novelty of the study is that for the first time, an increase in circulating SIRT2 is reported in PD and correlated with circulating α-Syn. The cut-off value, sensitivity and specificity of increased circulating SIRT2 obtained from the ROC analysis allowed us to distinguish PD from GC as well as APS. Its correlation with α-Syn suggests its association with early events in the pathogenesis of PD. It can be concluded from this study that SIRT2 may be a diagnostic marker for PD. A larger sample of PD as well as APSs, the use of CSF and follow-up assessment could provide further information on SIRT2 as a biomarker of PD.

## Limitation

The limitation of the present study is its cross-sectional nature and small APS sample size.

## Data Availability

No datasets were generated or analyzed for this study.

## Ethics Statement

The study was approved by the Ethics Committee, All India Institute of Medical Sciences, New Delhi, India (IECNP-221/05.06.2015, RP-01/2015) and written consent was obtained from each participant.

## Author Contributions

AS did major part of the experiment. GR, AD and MB provided all blood samples after diagnosis of the disease. VS and SDw performed the statistical analysis. TB was involved in α-synuclein purification. SDe conceptualized the research and wrote the manuscript. All authors read and approved the final manuscript.

## Conflict of Interest Statement

The authors declare that the research was conducted in the absence of any commercial or financial relationships that could be construed as a potential conflict of interest.

## Supplementary Material

FIGURE S1 Immobilization profile of **(A)** SIRT1, **(B)** SIRT2 and **(C)** α-syn antibody on CM5 sensor chip. Standard curve plotted between Resonance unit (RU) and known concentrations of purified recombinant **(D)** SIRT1 **(E)**, SIRT2 and **(F)** α-Syn.

FIGURE S2 **(A)** Scatter plot by independent *t*-test for serum α-Syn between PD vs. GC and **(B)** ROC curves of serum α-Syn PD vs. GC.
