# Supplementary figures and images for: Elevated Serum SIRT 2 May Differentiate Parkinson’s Disease From Atypical Parkinsonian Syndromes
Source: Front Mol Neurosci. 2019 Jun 12;12:129. doi: 10.3389/fnmol.2019.00129 (PMC6581755; doi:10.3389/fnmol.2019.00129)

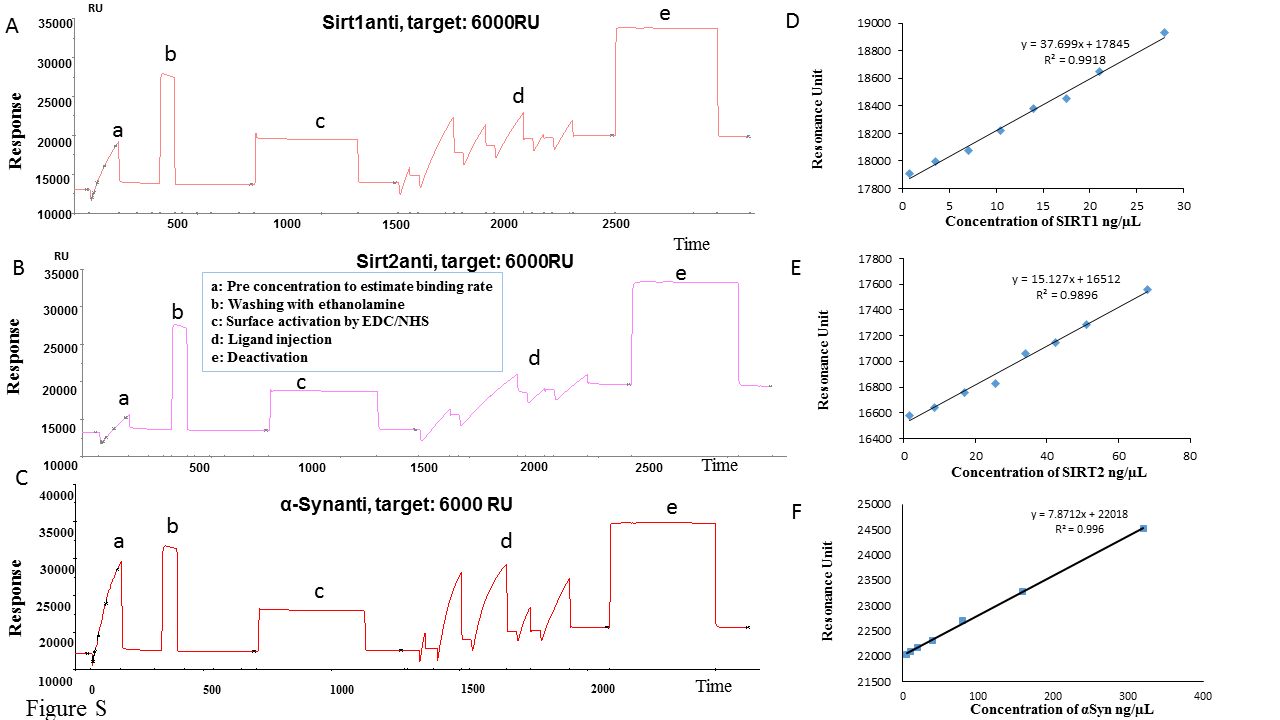

Supplement: FIGURE S1 — Immobilization profile of (A) SIRT1, (B) SIRT2 and (C) α-syn antibody on CM5 sensor chip. Standard curve plotted between Resonance unit (RU) and known concentrations of purified recombinant (D) SIRT1 (E), SIRT2 and (F) α-Syn. [file Image_1.tif]

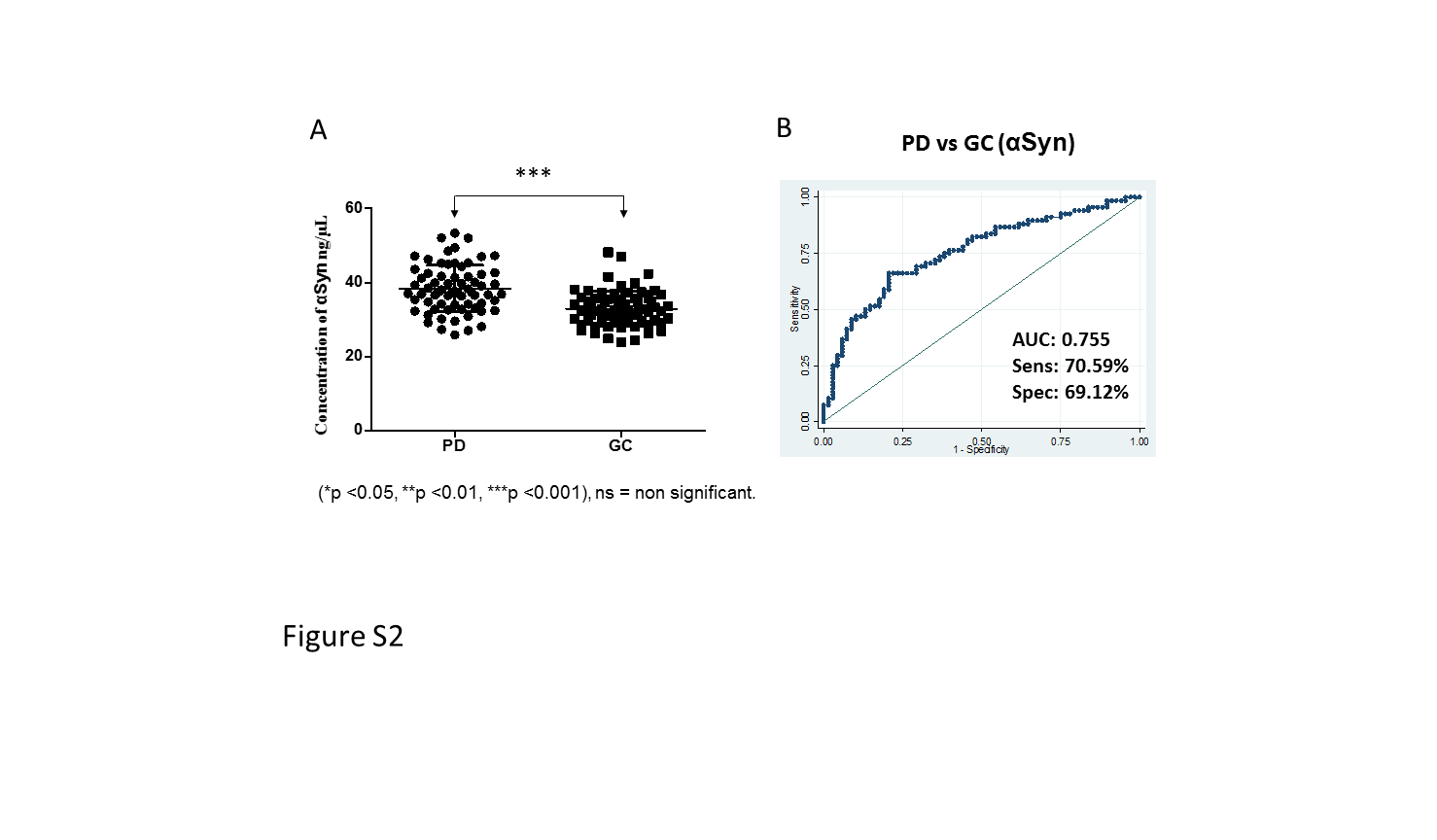

Supplement: FIGURE S2 — (A) Scatter plot by independent t-test for serum α-Syn between PD vs. GC and (B) ROC curves of serum α-Syn PD vs. GC. [file Image_2.TIF]
